# Supplementary material for: Association between oral microbiome and seven types of cancers in East Asian population: a two-sample Mendelian randomization analysis
Source: Front Mol Biosci. 2023 Nov 21;10:1327893. doi: 10.3389/fmolb.2023.1327893 (PMC10702768; doi:10.3389/fmolb.2023.1327893)
Supplement: Supplementary file 1 [file DataSheet1.ZIP › Supplementary Materials/MR plots of saliva/saliva═╝/Prostate cancer/pheno.278_to_prostate cancer_leave_one_out.pdf]

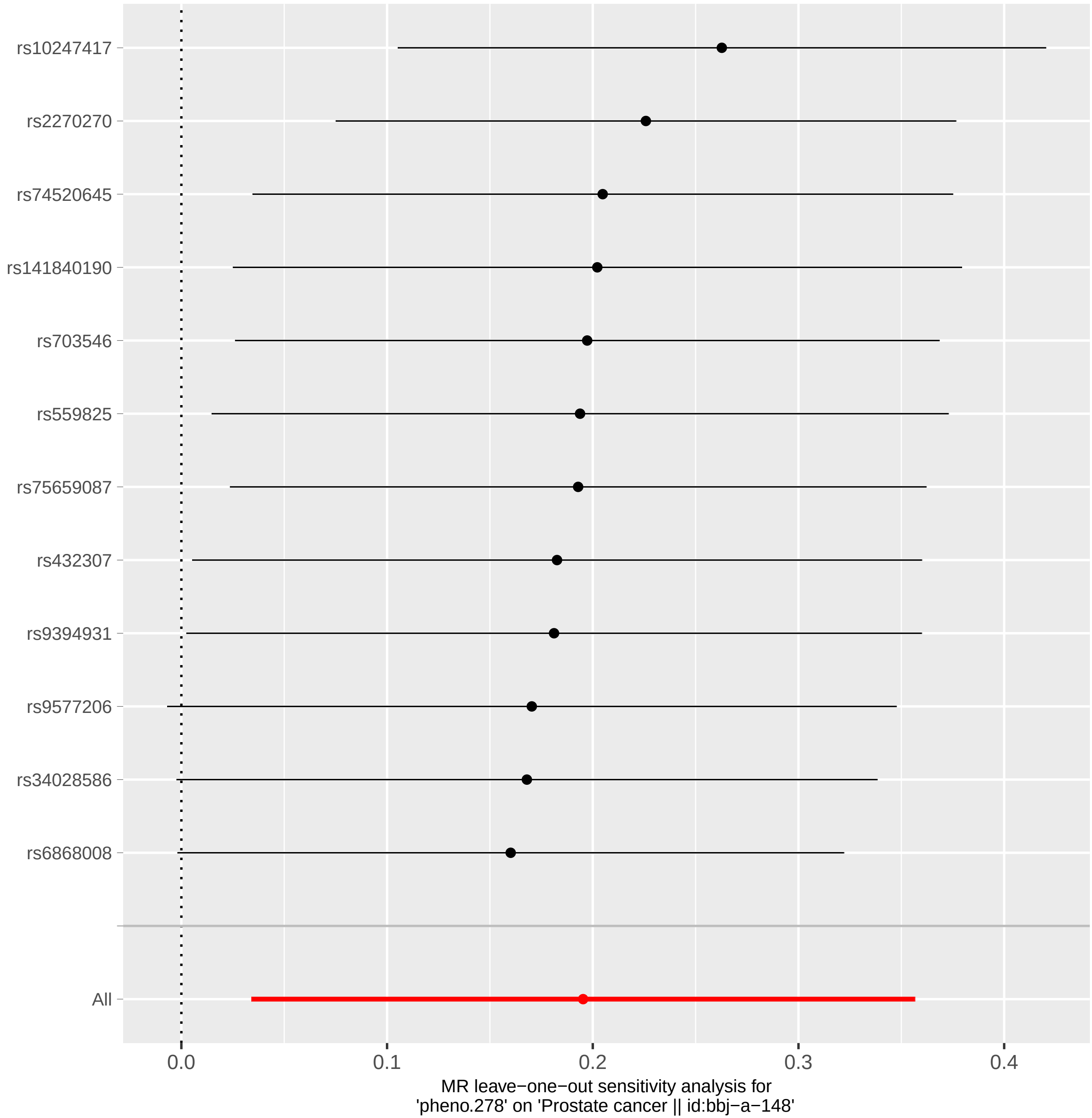
rs10247417
rs2270270
rs74520645
rs141840190
rs703546
rs559825
rs75659087
rs432307
rs9394931
rs9577206
rs34028586
rs6868008
All
0.0
0.1
0.2
0.3
0.4
MR leave−one−out sensitivity analysis for
'pheno.278' on 'Prostate cancer || id:bbj−a−148'
